# *FOXO3* Longevity Genotype Mitigates Risk Posed by Hypertension on Incident Coronary Artery Disease in Middle-Aged Men: Kuakini Honolulu Heart Program

**DOI:** 10.1093/gerona/glae254

**Published:** 2024-11-05

**Authors:** Randi Chen, Brian J Morris, Timothy A Donlon, Kazuma Nakagawa, Richard C Allsopp, Bradley J Willcox, Kamal H Masaki

**Affiliations:** Department of Research, Kuakini Honolulu Heart Program, Center of Biomedical Research Excellence (COBRE) for Clinical and Translational Research on Aging, Kuakini Medical Center, Honolulu, Hawaii; School of Medical Sciences, University of Sydney, Sydney, New South Wales, Australia; Department of Research, Kuakini Honolulu Heart Program, Center of Biomedical Research Excellence (COBRE) for Clinical and Translational Research on Aging, Kuakini Medical Center, Honolulu, Hawaii; Department of Cell and Molecular Biology, John A. Burns School of Medicine, University of Hawaii, Honolulu, Hawaii; Department of Geriatric Medicine, John A. Burns School of Medicine, University of Hawaii, Honolulu, Hawaii; Neuroscience Institute, The Queen’s Medical Center, Honolulu, Hawaii, USA; Department of Medicine, John A. Burns School of Medicine, University of Hawaii, Honolulu, Hawaii; Institute for Biogenesis Research, John A. Burns School of Medicine, University of Hawaii, Honolulu, Hawaii; Department of Research, Kuakini Honolulu Heart Program, Center of Biomedical Research Excellence (COBRE) for Clinical and Translational Research on Aging, Kuakini Medical Center, Honolulu, Hawaii; School of Medical Sciences, University of Sydney, Sydney, New South Wales, Australia; Department of Research, Kuakini Honolulu Heart Program, Center of Biomedical Research Excellence (COBRE) for Clinical and Translational Research on Aging, Kuakini Medical Center, Honolulu, Hawaii; School of Medical Sciences, University of Sydney, Sydney, New South Wales, Australia

**Keywords:** Coronary artery disease, *FOXO3*, Genetics, Hypertension, Longevity

## Abstract

**Background:**

This study tested whether the carriage of the longevity-associated *G*-allele of *FOXO3* SNP *rs2802292* (*TG*/*GG*) protects against incident coronary artery disease (CAD) in men with hypertension.

**Methods:**

Subjects were American men residing on Oahu having Japanese (*n* = 5415) or Okinawan (*n* = 897) ancestry and free of CAD at baseline (1965–1968) when aged 45–68 years.

**Results:**

During the follow-up, there were 1 629 incident CAD cases. Adjusting for age and cardiovascular disease risk factors, the main effect Cox model showed that in men of Japanese ancestry, hypertension was a strong predictor of CAD (hazard ratio [HR] 1.61; 95% confidence interval [CI] 1.44–1.80), *p* < .0001), but *TG*/*GG* genotype was not associated with CAD (HR 0.92; 95% CI = 0.82–1.02; *p* = .11). A full Cox model showed the interaction of *TG*/*GG* with hypertension was significant (β = –0.23, *p* = .038). Stratified by hypertension status, *TG*/*GG* genotype *TG*/*GG* had a protective effect against CAD in each group (HR 0.83; 95% CI 0.71–0.96; *p* = .021 in men of Japanese heritage, and HR 0.66; 95% CI 0.43–1.01; *p* = .054 in men of Okinawan heritage). No association with CAD was seen in normotensive men having either Japanese (HR 1.04; 95% CI 0.89–1.22; *p* = .61) or Okinawan (HR 0.95; 95% CI 0.66–1.38; *p* = .79) heritage.

**Conclusions:**

The present prospective study found that longevity-associated *FOXO3* genotype did not independently affect the risk of CAD in all men. Rather, it was associated with protection against incident CAD in men with hypertension. Hypertensive middle-aged men with *FOXO3TT* genotype may merit particular attention in CAD prevention programs.

## Graphical Abstract

**Graphical Abstract:**
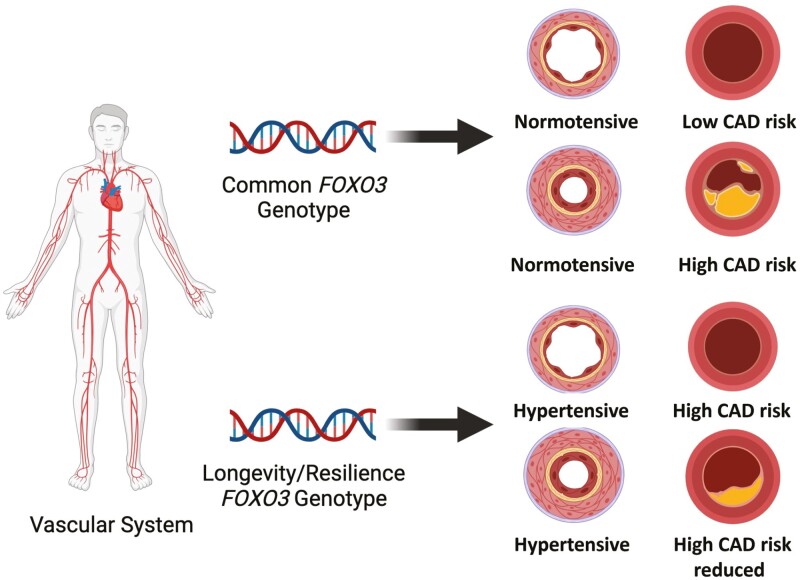

Coronary artery disease (CAD) is the leading cause of death in men and women globally. One of the major risk factors for CAD is hypertension. Although the incidence of each has a genetic component and gene variants associated with CAD and hypertension are known, little or nothing is known about genetic factors that fully account for (mediate) or partially account for (moderate) the influence of hypertension on the risk of CAD.

The present study focused on the well-established longevity-associated gene *FOXO3*, which encodes the ubiquitous forkhead winged-helix box O type 3 transcription factor, FOXO3. (Readers should note that the italicized symbol refers to the gene and the plain text symbol refers to the protein.)

FOXO3 (FoxO3 in rodents), by targeting promoters in genes across multiple tissues, controls numerous pathways that regulate mechanisms that enhance resilience to aging-related organismal stresses, thereby slowing the aging process and increasing longevity (1,2). The pathways include mitigation of oxidative stress, enhancement of stem-cell maintenance, DNA damage repair, as well as modulation of apoptosis, autophagy, mitophagy, lipophagy, and redox balance, thus resulting in enhanced stress resistance (2). Cardiovascular aging is often intimately tied to aging-related health deterioration (3). Some of the pathways mediate the protection that FOXO3 exerts on cardiovascular aging, specifically by the suppression of vascular smooth muscle cell proliferation and neointimal hyperplasia, maintaining human vascular cell homeostasis, counteracting atherosclerosis, slowing down vascular and organismal aging, increasing resistance to oxidative injury, promoting homeostasis of a diverse array of vascular cell types, and mitigating cardiac hypertrophy (4).

The longevity genotype of *FOXO3* single nucleotide polymorphism (SNP) *rs2802292* has shown a resilience effect on mortality in response to stress in those who have a cardiometabolic disease (namely, hypertension, prevalent CAD, and/or diabetes) (5). Individuals who are carriers of the minor allele (*G*; genotype *TG*/*GG*) have on average longer lifespan, whereas major allele homozygotes (*TT* genotype) are more likely to have an average lifespan. Proteomics analyses have revealed specific proteins associated with the amelioration of chronic biological stress in response to the *FOXO3* longevity genotype in our cohort (6). Stress factors that lead to hypertension also lead to other diseases. Based on our previous findings and new knowledge, we proposed that *FOXO3* longevity genotype confers cellular and organismal resilience via the following pathways: (a) response to innate immunity, (b) response to inflammation, (c) moderation of signal transduction, (d) moderation of growth factor levels, and (e) maintenance of stem cells. These may confer protection against the adverse effects of hypertension.

Recently, we found that the longevity genotype of *FOXO3* moderated the increased risk of late-life hypertension in Alzheimer’s disease (7), attenuated the deleterious impact of chronic hypertension on the long-term risk of spontaneous intracerebral hemorrhage, possibly by providing cerebrovascular resilience and protection against the adverse effects of chronic hypertension (8), and attenuated the impact of hypertension on the risk of cerebral microinfarcts identified in autopsies of deceased individuals in our cohort (9).

The aim of the present study was to test the hypothesis that the longevity genotype of *FOXO3* mitigates the risk posed by hypertension on the incidence of CAD in our cohort of middle-aged American men with traditional Japanese ancestry and to perform a replication study in a genetically distinct sample of American men of Okinawan ancestry.

## Materials and Methods

### Study Design

The Kuakini Honolulu Heart Program (Kuakini HHP) is a prospective population-based longitudinal study of cardiovascular disease (CVD). Procedures performed were in accordance with institutional guidelines and written informed consent was obtained at all examination cycles. The study was approved by the Institutional Review Board of Kuakini Medical Center.

### Participants

Eight thousand and six men aged 45–68 years, resident on the island of Oahu, HI, and who were of traditional Japanese ancestry and Okinawan ancestry, were recruited in 1965–1968 (10–12). All were drawn from World War II Selective Service Registration files as described previously (13). The men were then followed with periodic examinations and continuous surveillance of hospital records for the development of CAD, stroke, and other selected morbidity, and mortality through December 31, 2000. Very little attrition occurred, with only 5 men having been lost to follow-up by the fourth examination. The men had immigrated to Hawaii from Japan and from the island of Okinawa in the early 20th century. Okinawa has been a longstanding population isolate genetically more similar to populations of East Asia (China and Korea) than Japan, which annexed it in 1879 (14). Therefore, the Kuakini HHP cohort comprised 2 genetically (and culturally) different population-based cohorts, that is, the Japanese-ancestry cohort and the Okinawan-ancestry cohort.

### Data Collection Methods

Blood pressure (BP) was measured in the left arm in the seated position using a standardized protocol (15), twice by a trained research nurse and once by a physician, after at least 10 minutes of rest. The mean value of all available BP measurements was used. Hypertension at baseline (Kuakini HHP examination 1 in 1965–1968) was defined as systolic BP ≥140 mm Hg and/or diastolic BP ≥90 mm Hg, or the self-reported use of antihypertensive medications. Being normotensive was defined as having a systolic BP <140 mm Hg and diastolic BP <90 mm Hg, and not taking antihypertensive medication.

Body mass index (BMI) was defined as weight in kilograms divided by height in meters squared. Physical activity index was the overall metabolic output in a 24-hour period, calculated by multiplying the approximate oxygen consumption of 5 different activity levels (no activity = 1.0, sedentary = 1.1, slight = 1.5, moderate = 2.4, heavy = 5.0) with self-reported hours per day spent in performing these activities (16). Serum glucose and cholesterol levels were measured in blood samples collected 1 hour after oral administration of a 50-g glucose load. Alcohol intake (ounces per month) was computed from self-report. Smoking was defined as pack-years by self-report. Diabetes was based on self-report.

### Genotyping

Genotyping of *FOXO3* SNP *rs2802292* was performed using DNA from the buffy coat of blood samples collected at Kuakini HHP Exam 4 (1991–1993) and that had been frozen at –70°C (17). For those who did not attend Exam 4 (meaning we did not have blood samples from those men), we used their blood serum samples available from Exam 3 in 1971–1974. DNA was obtained from the serum samples using a combination of QIAmp cell-free DNA isolation followed by REPLI-g Single-Cell WGA & WTA amplification (QIAGEN Sciences, Germantown, MD). Genotyping was performed using TaqMan on an Applied Biosystems QuantStudio 12K Flex system (ThermoFisher Scientific, Waltham, MA). In 2020, *FOXO3* SNP *rs2802292* genotyping was completed for all men who had provided blood samples at either Exam 3 or Exam 4. We have found that our *FOXO3* genotyping methods are exceptionally robust, have an accuracy of >99.9% on retesting, and a success rate of 99.6% (data not shown).

### Incident Coronary Artery Disease

Incident CAD was diagnosed by an expert physician Morbidity and Mortality committee using standard research criteria. This committee reviewed all hospital records for heart disease hospitalizations through to December 31, 2000, when Kuakini HHP surveillance on cardiovascular outcomes ceased (35 years of follow-up). The date of the first coronary event was defined as the onset date of CAD. Coronary events included myocardial infarction (defined as an acute or temporal change on electrocardiogram (ECG) or elevation in cardiac enzyme levels), coronary insufficiency (severe coronary-type chest pain lasting 30 minutes or longer with ECG evidence of transient ST-T changes), angina, coronary artery bypass graft, coronary angioplasty, silent myocardial ischemia (ECG temporal changes suggestive of MI from hospital records or follow-up examinations—Exam 2, Exam 3, Exam 4, Exam 5), and CAD death (sudden death within an hour of observation that could not be attributed to another cause and with typical features of angina, pathological findings of acute infarct at autopsy or surgery, or decompensated heart failure leading to death).

### Statistical Analyses

Mean and percentage of baseline risk factor levels were compared among subjects according to midlife hypertension status and *FOXO3* genotype (*TT* or *TG*/*GG*) using *t*-tests for means of continuous variables, and chi-squared tests for binary variables. Kaplan–Meier disease-free survival curves and Cox proportional hazard models were used to assess the association of *rs2802292* genotype and hypertension with incident CAD. We analyzed the data from 3 different subsets: participants originating from the historical islands of Japan, participants originating from Okinawa, and both of these combined. The Japanese-ancestry cohort was the test sample, and the Okinawan-ancestry cohort was the replication sample. A main effect Cox model included both main factors, that is, *FOXO3* genotype and hypertension status, adjusting for baseline age and CAD risk factors: BMI, total cholesterol, smoking (pack-years), physical activity, glucose, alcohol consumption (oz/month), and prevalent diabetes. A full Cox model included the main factors (*FOXO3* genotype and hypertension status), and the interaction term of *FOXO3* genotype and hypertension status, adjusting for age and CVD risk factors. Proportional hazard assumptions of hypertension status and *FOXO3* genotypes on CAD were examined using a log cumulative hazard plot.

## Results

In this longitudinal prospective study of 8 006 middle-aged men in the Kuakini HHP cohort, to ensure only men free of CAD at baseline were included, we excluded 390 prevalent CAD cases at Exam 1 in 1965–1968. We also excluded 1 304 men without *FOXO3* genotype data, because they either did not participate or did not have blood samples available from Kuakini HHP Exam 3 or 4. The final analytical sample for our study comprised 6 312 men. Of these, there were 5 415 men of traditional Japanese ancestry and 897 men of Okinawan ancestry. In the follow-up period of 35 years to December 31, 2000, 1 629 incident CAD cases were diagnosed. Of the 1 629 CAD cases, 187 were diagnosed as CAD cases based on the cause of death being from CAD (11.5% of the 1 629 CAD cases).

Table 1 shows the mean and percentage of baseline characteristics of the 2 cohorts. Compared to men originating from the traditional islands of Japan, Okinawan men had higher levels of BMI, physical activity and alcohol intake, and less pack-years smoked and diabetes. However, no significant differences were found in hypertension status and *rs2802292* longevity genotype *TG*/*GG* between men of traditional Japanese ancestry and those of Okinawan ancestry.

**Table 1.** Baseline Characteristics

	Japanese-Ancestry Cohort (*n* = 5 415)	Okinawan-Ancestry Cohort (*n* = 897)	
Continuous variables	Mean (*SD*)	Mean (*SD*)	*p* Value
Age, years	54.1 (5.4)	52.9 (.3)	<.0001
BMI (kg/m^2^)	23.8 (3.0)	24.5 (3.0)	<.0001
Physical activity index	32.7 (4.5)	33.8 (4.7)	<.0001
Smoking, pack-years	22.8 (23.9)	21.1 (22.2)	.041
Alcohol intake, oz/month	13.2 (23.2)	15.3 (25.9)	.016
Post-load glucose, mg/dL	158.9 (54.8)	155.6 (57.0)	.099
Total cholesterol, mg/d	217.8 (36.5)	219.5 (38.5)	.20
Binary variables	*n* (%)	*n* (%)	*p* Value
Hypertension			
No	3 290 (60.8)	549 (61.2)	.80
Yes	2 125 (39.2)	348 (38.8)	
Prevalence of diabetes			
No	4 911 (90.9)	843 (93.0)	.046
Yes	489 (9.1)	63 (7.0)	
Missing	15	0	
*FOXO3 genotype*			
* TT*	2 884 (53.3)	494 (55.1)	.31
* TG/GG*	2 531 (46.7)	403 (44.9)	

*Notes*: BMI = body mass index; *SD* = standard deviation.

Table 2 shows the age-adjusted incidence of CAD (number of CAD cases per 1 000 person-years) for *FOXO3* longevity genotypes in the Japanese-ancestry cohort (the test sample) with and without hypertension. The incidence of CAD was higher in subjects with hypertension compared with those who were normotensive (*p* < .0001; Table 2, column A). There was, however, no significant difference in CAD incidence between the *FOXO3 TG*/*GG* and *TT* genotypes (*p* = .30; Table 2, column B). Among normotensive subjects, CAD incidence did not differ significantly between *FOXO3* genotypes (*p *= .59; Table 2, row C). In contrast, among men with hypertension, those with the *FOXO3* longevity genotype (*TG*/*GG*) had significantly lower CAD incidence compared with those having the *FOXO3 TT* genotype (*p* = .037; Table 2, row C). The stratified differences suggested an interaction effect between *FOXO3* genotype and hypertension status in relation to CAD incidence.

**Table 2.** Association of *FOXO3* Longevity Genotypes With Incident CAD by Hypertension Status for Japanese-Ancestry Cohort

	A. Hypertension Status			B. *FOXO3* Genotypes		
	NT	HTN	*p* Value	*TT*	*TG*/*GG*	*p* Value
*n* (CAD)	3 290 (699)	2 125 (716)		2 884 (769)	2 531 (646)	
CAD cases per 1 000 person-years	6.92	12.06	<.0001	9.00	8.55	.30
C. *FOXO3* Genotypes, Stratified by Hypertension Status						
	NT			HTN		
	*TT*	*TG*/*GG*	*p* Value	*TT*	*TG*/*GG*	*p* Value
*n* (CAD)	1 756 (366)	1 534 (333)		1128 (403)	997 (313)	
CAD cases per 1 000 person-years	6.80	7.07	0.59	12.91	10.97	0.037

*Notes*: CAD = coronary artery disease; HTN = hypertensive; NT = normotensive.

**Figure 1.**
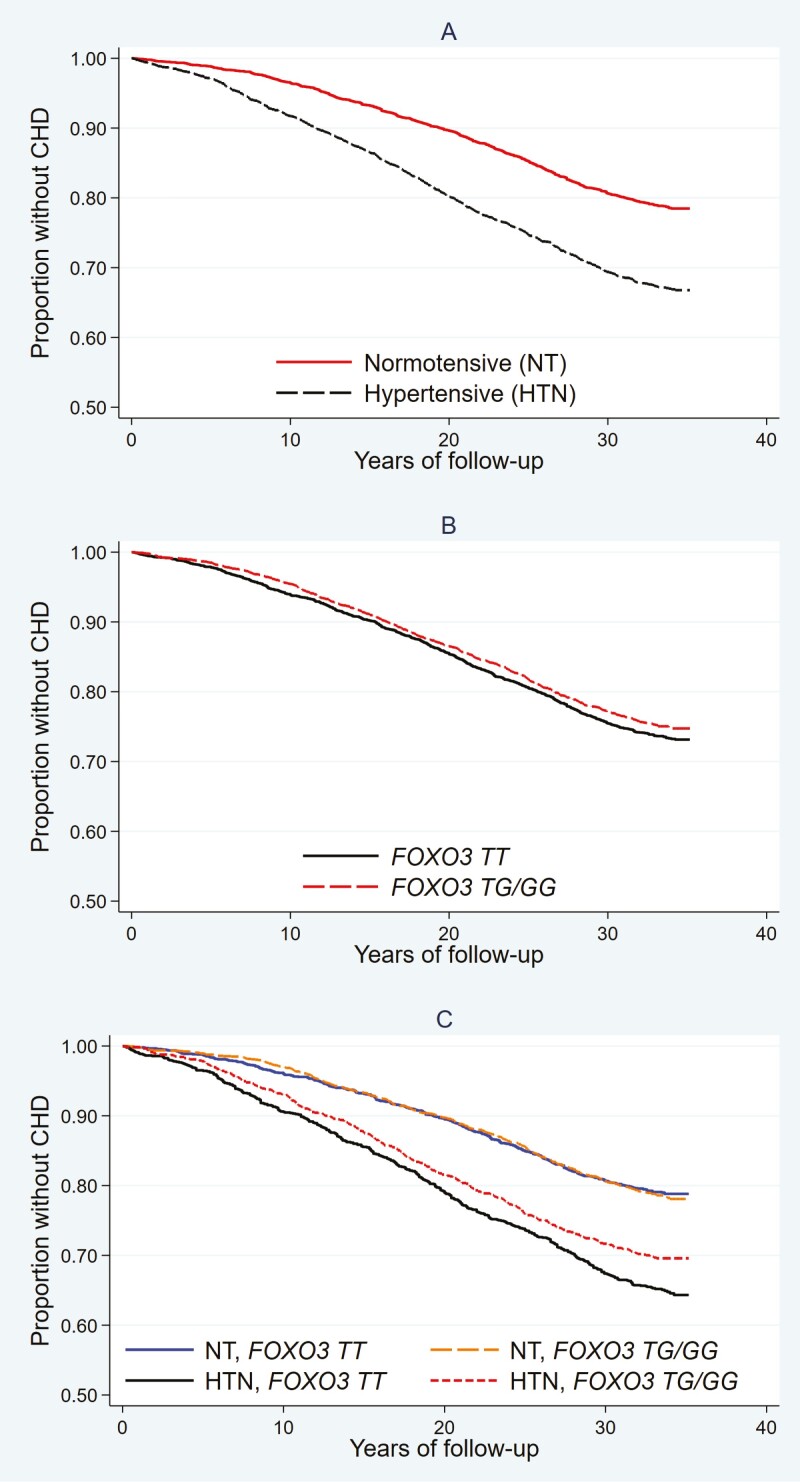
Kaplan–Meier survival curves for cohort participants free of incident coronary artery disease (CAD) by hypertension status, by *FOXO3* genotypes, and by different combinations of hypertension status and *FOXO3* genotypes for Japanese-ancestry cohort. The *p-*values of log rank tests are, for part A (HTN vs NT), *p = *5.8 × 10^–27^, for part B (*FOXO3 TT* vs *TG*/*GG* genotype), *p* = .14, and for part C (HTN × *FOXO3* genotype) comparing genotype *FOXO3 TG/GG* vs *TT, p* = .75 in NT, and *p* = .013 in HTN; comparing HTN vs NT, *p* = 9.6 × 10^–9^ for *FOXO3* genotype *TG/GG*, and *p* = 5.7 × 10^–21^ for *FOXO3* genotype *TT*. HTN, hypertensive; NT, normotensive.

Figure 1 shows Kaplan–Meier curves for survival free of incident CAD by hypertension status and by *FOXO3* genotypes, as well as by different combinations of hypertension status and *FOXO3* genotypes for the Japanese-ancestry cohort. It further shows an interaction effect between hypertension status and *FOXO3* genotype on CAD incidence in the cohort.

Table 3 shows the results of the main effect Cox models and full Cox models for cohorts of the Japanese-ancestry, Okinawan-ancestry, and the Kuakini HHP cohort. In the main effect Cox models adjusting for age and baseline CAD risk factors as potential confounders, hypertension was a highly significant risk factor for CAD incidence in the Japanese-ancestry cohort and the Kuakini HHP cohort, but not in the Okinawan-ancestry cohort, possibly due to the small sample size. *FOXO3* genotype was not associated with CAD in all 3 cohorts. The significant likelihood ratio test showed that the full model fitted data better than the main effect model; therefore, the full model was used to interpret the data.

**Table 3.** Cox Regression Models for Main Effect of Hypertension and *FOXO3* Genotype, and Interaction Effect of Hypertension With *FOXO3* Genotype on Incident CAD

	Hypertension		*TG*/*GG*		
	HR (95% CI)^*^	*p* Value	HR (95% CI)	*p* Value	α for Interaction (*p* Value)^†^
Japanese-ancestry cohort (*n* = 5 415)	1.61 (1.44–1.80)	<.0001	0.92 (0.82–1.02)	.11	–0.23 (.038)
Okinawan-ancestry cohort (*n* = 897)	1.15 (0.86–1.55)	.34	0.82 (0.62–1.08)	.15	–0.37 (.20)
Entire Kuakini HHP cohort (*n* = 6 312)	1.54 (1.39–1.71)	<.0001	0.91 (0.82–1.00)	.053	–0.23 (.026)

*Notes*: BMI = body mass index; CAD = coronary artery disease; CI = confidence interval; HR = hazard ratio; Kuakini HHP = Kuakini Honolulu Heart Program.

^*^HRs, 95% CI, and interaction coefficients estimated from Cox proportional hazards models adjusting for age, BMI, total cholesterol, smoking (pack-years), physical activity, glucose, alcohol consumption (oz/month), and prevalent diabetes when including both hypertension and *FOXO3 TG*/*GG* genotype as main effects.

^†^Estimated from the full Cox model, adding the interaction term of hypertension and *FOXO3 TG*/*GG* genotype to the main effect model.

The significant interaction of hypertension with *FOXO3* genotype in the Japanese-ancestry cohort and the Kuakini HHP cohort in the full models indicated that the effect of *FOXO3* genotype on incident CAD differed between subjects with and without hypertension. We next performed Cox models adjusting for age and CVD risk factors stratified by hypertension status for the 3 cohorts (Table 4). In Table 4, row A, where we treated CAD deaths as incident CAD cases, we found that among normotensive subjects, there was no association of *FOXO3* genotype (*TG*/*GG* vs *TT*) with incident CAD estimated from Cox modeling (HR range = 0.95–1.01; *p* > .70 for all 3 samples). In contrast, among subjects with hypertension, *FOXO3 TG*/*GG* genotype was associated with an apparent protective effect against incident CAD compared to the major allele homozygote *TT* (HR = 0.83, *p* = .014, for Japanese-ancestry cohort; HR = 0.66, *p* = .054, for Okinawan-ancestry cohort; and HR = 0.82, *p* = .0053, for the Kuakini HHP cohort). Of note, in the Okinawan-ancestry cohort, the protective effect of *FOXO3 TG*/*GG* genotype on CAD (HR = 0.66; *p* = .054) was greater than that in the Japanese-ancestry cohort (HR = 0.83), although significance was borderline, most likely due to the small sample size. Similar effects of *FOXO3 TG*/*GG* genotype on incident CAD, as in Table 4, row A, were seen in models in which we excluded CAD deaths from the analyses (Table 4, row B).

**Table 4.** Hazard Ratio of *FOXO3* Genotype *TG*/*GG* Versus *TT* for Incident CAD, Stratified by Hypertension Status

		Normotensive			Hypertensive		
A	Cohort	*n* (CAD)^*^	HR (95% CI)^†^	*p* Value	*n* (CAD)	HR (95% CI)	*p* Value
	Japanese-ancestry cohort	3 290 (699)	1.03 (0.88–1.19)	.74	2 125 (716)	0.83 (0.71–0.96)	.014
	Okinawan-ancestry cohort	549 (117)	0.95 (0.66–1.38)	.79	348 (97)	0.66 (0.43–1.01)	.054
	Kuakini HHP cohort	3 839 (816)	1.01 (0.88–1.17)	.85	2 473 (813)	0.82 (0.71–0.94)	.0053
B	Cohort	*n* (CAD)	HR (95% CI)	*p* Value	*n* (CAD)	HR (95% CI)	*p* Value
	Japanese-ancestry cohort	3 223 (632)	1.04 (0.89–1.22)	.61	2 032 (623)	0.83 (0.70–0.97)	.021
	Okinawan-ancestry cohort	541 (109)	0.94 (0.60–1.30)	.54	329 (78)	0.63 (0.39–1.01)	.055
	Kuakini HHP cohort	3 764 (741)	1.02 (0.88–1.18)	.82	2 361 (701)	0.82 (0.70–0.95)	.0086

*Notes*: BMI = body mass index; CAD = coronary artery disease; CI = confidence interval; HR = hazard ratio; Kuakini HHP = Kuakini Honolulu Heart Program. (A) When CAD death was included in the definition of CAD incidence. (B) When CAD death was excluded from the analyses.

^*^Total number of subjects and (in brackets) number with incident coronary heart disease (CAD).

^†^HR and 95% CI estimated from Cox proportional hazard models adjusting for baseline age, BMI, total cholesterol, smoking (pack-years), physical activity, glucose, alcohol consumption (oz/month), and prevalent diabetes.

In addition, we performed similar Cox models stratified by *FOXO3* genotypes for the 3 samples (Table 5). In Table 5, row A, where we treated CAD-deaths as incident CAD cases, hypertension remained a strong risk factor for incident CAD in all groups, but the HRs were lower in the *TG*/*GG* group, suggesting that the longevity genotype attenuates the risk posed by hypertension on incident CAD. Similar effects of hypertension on incident CAD, as in Table 5, row B, were seen in models in which we excluded CAD deaths from the analyses.

**Table 5.** Hazard Ratio of HTN Versus NT for Incident CAD, Stratified by *FOXO3* Longevity Genotypes

		*TT*			*TG*/*GG*		
A	Cohort	*n* (CAD)^*^	HR (95% CI)^†^	*p* Value	*n* (CAD)	HR (95% CI)	*p* Value
Japanese-ancestry cohort	2 884 (769)	1.95 (1.69–2.25)	<.0001	2 531 (646)	1.58 (1.36–1.85)	<.0001
Okinawan-ancestry cohort	494 (127)	1.53 (1.08–2.17)	.016	403 (87)	1.21 (0.79–1.87)	.38
Kuakini HHP cohort	3 378 (896)	1.87 (1.64–2.14)	<.0001	2 934 (733)	1.53 (1.32–1.77)	<.0001

B	Cohort	*n* (CAD)	HR (95% CI)	*p* Value	*n* (CAD)	HR (95% CI)	*p* Value
Japanese-ancestry cohort	2 792 (677)	1.78 (1.51–2.09)	<.0001	2 463 (578)	1.4 (1.18–1.67)	.0002
Okinawan-ancestry cohort	480 (113)	1.14 (0.77–1.69)	.52	390 (74)	0.78 (0.45–1.33)	.36
Kuakini HHP cohort	3 272 (790)	1.66 (1.43–-1.93)	<.0001	2 853 (652)	1.33 (1.13–1.57)	.0008

*Notes*: BMI = body mass index; CAD = coronary artery disease; CI = confidence interval; HR = hazard ratio; Kuakini HHP = Kuakini Honolulu Heart Program. (A) CAD death included in definition of CAD incidence. (B) CAD death excluded.

^*^Total number of subjects and (in brackets) number with CAD.

^†^HR and 95% CI were estimated from Cox proportional hazards models adjusting for baseline age, BMI, total cholesterol, smoking (pack-years), physical activity, glucose, alcohol consumption (oz/month), and prevalent diabetes.

## Discussion

The present longitudinal study of the Kuakini HHP cohort of American men of Japanese ancestry found no primary association of *FOXO3* genotype with the incidence of CAD. In contrast, in subjects with hypertension, a strong association was found between *FOXO3 G*-allele carriage and protection against incident CAD. No protective effect of *TG*/*GG* genotype against incident CAD was evident in normotensive subjects. This association was the same for men of Japanese ancestry and Okinawan ancestry.

In previous studies, we reported a protective effect of *FOXO3* longevity genotype on CAD mortality (18,19). Because we included deaths from CAD in the definition of incident CAD in the present study, we wondered whether CAD deaths might be contributing to the current results. However, the sensitivity analyses showed when CAD deaths, which accounted for 11.5% percent of incident CAD cases, were removed from the analysis, the effect of *FOXO3* genotype in moderating the effect of hypertension on incident CAD remained unchanged (Tables 4, row B and 5, row B).

The findings in the present study may have general significance to genetic studies of aging-related diseases. To be more specific, the inclusion of interaction between a gene variant and a risk factor such as hypertension could be crucial when studying a genetic relationship with an aging-related disease. In the present study, a finding that longevity/resilience-related *FOXO3* genotypes of this well-known longevity gene may have a moderating effect in protecting against the increased risk of CAD posed by hypertension could help explain how this genotype contributes, at least in part, to amelioration of aging-related disease risk.

Hypertension is a well-established risk factor for CAD because it causes structural, functional, and neurohormonal abnormalities in the heart (20). The myocardial remodeling that occurs leads to the development of left ventricular hypertrophy, myocardial and vascular fibrosis, microstructural changes, as well as disturbances in heart rhythm and conduction (20). High BP leads to changes in macrostructure, and left ventricular hypertrophy increases the risk of cardiac arrythmias in hypertensive patients, hypertension being the most important predictor of heart failure and sudden death (21). Myocardial ischemia and left atrial remodeling lead to atrial fibrillation, stroke, myocardial infarction, and mortality (21). In addition, the mechanical stress on the coronary artery endothelium from chronic hypertension promotes atherosclerosis and plaque formation, and ultimately plaque rupture leading to myocardial infarction (4). We, therefore, propose that elevation in intracellular FOXO3 levels, as particularly evident for *FOXO3 G*-allele carriers (22), may protect against progressive injuries to the coronary arteries of individuals with chronic hypertension, thereby protecting them from CAD-associated clinical manifestations.

The mechanism responsible for the effect of the longevity-associated *FOXO3* variant in our study on CAD risk in hypertensive patients may involve a number of factors. As explained in first section, FOXO3 is well known to counteract numerous adverse pathways that damage cells throughout the body (1–4). Arterial and arteriolar cell pathology is involved in the etiology of CAD and hypertension. This involves coronary artery occlusion and an increase in peripheral resistance from vascular constriction. FOXO3, and thus its protective effects, is downregulated in vascular cells during primate aging (23). Fundamental to the actions of FOXO3 is its ability to counteract age- and disease-related inflammation (1,24). In a previous article in this journal, we found the *FOXO3G*-allele was associated with a reduction in inflammatory markers TNF-α and CRP in our cohort (19). FOXO3 is a key regulator of stem-cell maintenance in many tissues, enhancing stress resistance stem-cell survival (25). By targeting the granulocyte/monocyte progenitor compartment in LDL receptor knockout mice, FOXO3 ameliorates atherosclerotic lesion formation (26). FOXO3-engineered embryonic stem cell–derived vascular cells have been shown to (i) improve vascular homeostasis and delay vascular aging (4), and (ii) promote vascular regeneration in response to ischemic injury (4,27). In addition, FOXO3 integrates the pleiotropic actions of insulin to protect the vascular endothelium from atherosclerosis (28). Regulating apoptosis of vascular smooth muscle cells is another means, whereby FOXO3 protects against atherosclerosis (29). The increased apoptosis and extracellular matrix breakdown in vascular smooth muscle induced by FOXO3 activation processes are mediated by matrix metalloproteinase 13 (30).

Our findings support the feasibility of stratifying middle-aged men by *FOXO3* genotype and hypertension status in assessing their risk of CAD. Furthermore, it may be worthwhile formulating a *FOXO3* genotype- and hypertension-specific CAD prevention program to reduce incident CAD, specifically in middle-aged men with the *FOXO3 TT* genotype.

A limitation of our study was that it was confined to American men of traditional Japanese ancestry, and of genetically distinct Okinawan ancestry, living in Hawaii. Consequently, we cannot be sure of the generalizability of the study findings to other ethnic groups. In contrast to men, among women in Japan, the longevity-associated *G*-allele of *rs2802292* is associated with increased CAD prevalence (31). However, a strength of our study is that all men underwent the same follow-up and regular examinations so that genetic ancestry was the only differentiating factor. We nevertheless encourage studies of other Japanese cohorts and of other races and ethnic groups.

The study had a number of strengths. These included the large overall sample size, the longitudinal prospective study design used, the comprehensive surveillance system for ascertainment of outcomes, and the large number of relevant clinical and biological parameters that were monitored at each examination. Thus, all men underwent the same 35 years of follow-up and regular examinations. The men of Japanese and Okinawan descent studied were unique in that each population is genetically more homogenous than populations of other racial types (14). Asian populations have a higher degree of linkage disequilibrium between single nucleotide polymorphism (SNPs) compared to other races. Such populations may be more beneficial for finding genotype–disease associations that may be less common in other ethnic groups (32). Furthermore, the hypothesis we tested—namely a moderating effect of longevity/resilience genotypes of a gene associated with longevity between hypertension and incident CAD risk—has not, to the best of our knowledge, been tested previously. This includes other comparable Japanese populations. Moreover, we had a considerable amount of data on other cardiovascular risk factors, allowing us to adjust for these factors during statistical analyses using Cox models to minimize confounding. Our surveillance system for incident CAD was thorough, facilitated by ours being an island population, and follow-up was meticulous.

Our findings support the feasibility of stratifying middle-aged men by *FOXO3* genotype and hypertension status in assessing their risk of CAD. Furthermore, it may be worthwhile formulating a *FOXO3* genotype- and hypertension-specific CAD prevention program to reduce incident CAD, specifically in middle-aged men with the *FOXO3 TT* genotype.

The findings also have therapeutic implications. There is compelling evidence that pharmacological approaches that target FOXO3 should help in the treatment of cardiovascular and other diseases of aging (33). An extensive review compiled a list of the pharmaceuticals and natural compounds that activate FOXO3 (34). Testing of FOXO3 activators should progress from animal models to trials of efficacy in the treatment of CVD in humans, and ultimately to clinical availability for patients.

## Conclusion

The present study found that the *FOXO3* longevity-associated genotype strongly protects against incident CAD in men with hypertension. No association was found with incident CAD in normotensive men.

## References

[1] Morris BJ , Willcox DC, Donlon TA, Willcox BJ. *FOXO3*: a major gene for human longevity—a mini-review. Gerontology. 2015;61:515–525. https://doi.org/10.1159/000375235 25832544 PMC5403515

[2] Bernardo VS , Torres FF, da Silva DGH. FoxO3 and oxidative stress: a multifaceted role in cellular adaptation. J Mol Med. 2023;101:83–99. https://doi.org/10.1007/s00109-022-02281-5 36598531

[3] Abdellatif M , Rainer PP, Sedej S, Kroemer G. Hallmarks of cardiovascular ageing. Nat Rev Cardiol. 2023;20:754–777. https://doi.org/10.1038/s41569-023-00881-3 37193857

[4] Zhao Y , Liu YS. Longevity factor FOXO3: a key regulator in aging-related vascular diseases. Front Cardiovasc Med. 2021;8:778674. https://doi.org/10.3389/fcvm.2021.778674 35004893 PMC8733402

[5] Chen R , Morris BJ, Donlon TA, et al *FOXO3* longevity genotype mitigates the increased mortality risk in men with a cardiometabolic disease. Aging (Milano). 2020;12:23509–23524. https://doi.org/10.18632/aging.202175 PMC7762472 33260156

[6] Donlon TA , Morris BJ, Chen R, et al Proteomic basis of mortality resilience mediated by *FOXO3* longevity genotype. GeroScience. 2023;45:2303–2324. https://doi.org/10.1007/s11357-023-00740-6 36881352 PMC10651822

[7] Chen R , Morris BJ, Donlon TA, et al Incidence of Alzheimer’s disease in men with late-life hypertension is ameliorated by *FOXO3* longevity genotype. J Alzheimers Dis. 2023;95:79–91. https://doi.org/10.3233/JAD-230350 37483002 PMC10578238

[8] Nakagawa K , Chen R, Greenberg SM, et al Forkhead box O3 longevity genotype may attenuate the impact of hypertension on risk of intracerebral haemorrhage. J Hypertens. 2022;40:2230–2235. https://doi.org/10.1097/HJH.0000000000003249 35943066 PMC9553272

[9] Nakagawa K , Chen R, Ross GW, et al *FOXO3* longevity genotype attenuates the impact of hypertension on cerebral microinfarct risk. J Hypertens. 2024;42:484–489. https://doi.org/10.1097/HJH.0000000000003620 38009316 PMC10873049

[10] Kagan A , Harris BR, Winkelstein W, Jr, et al Epidemiologic studies of coronary heart disease and stroke in Japanese men living in Japan, Hawaii and California: demographic, physical, dietary and biochemical characteristics. J Chronic Dis. 1974;27:345–364. https://doi.org/10.1016/0021-9681(74)90014-9 4436426

[11] Yano K , Reed DM, McGee DL. Ten-year incidence of coronary heart disease in the Honolulu Heart Program. Relationship to biologic and lifestyle characteristics. Am J Epidemiol. 1984;119:653–666. https://doi.org/10.1093/oxfordjournals.aje.a113787 6720665

[12] Kagan A , ed. The Honolulu Heart Program: An Epidemiological Study of Coronary Heart Disease and Stroke. Harwood Academic Publishers; 1996.

[13] Worth RM , Kagan A. Ascertainment of men of Japanese ancestry in Hawaii through World War II Selective Service registration. J Chronic Dis. 1970;23:389–397. https://doi.org/10.1016/0021-9681(70)90022-6 5492969

[14] Bendjilali N , Hsueh WC, He Q, et al Who are the Okinawans? Ancestry, genome diversity, and implications for the genetic study of human longevity from a geographically isolated population. J Gerontol A Biol Sci Med Sci. 2014;69:1474–1484. https://doi.org/10.1093/gerona/glt203 24444611 PMC4271021

[15] Yano K , McGee D, Reed DM. The impact of elevated blood pressure upon 10-year mortality among Japanese men in Hawaii: the Honolulu Heart Program. J Chronic Dis. 1983;36:569–579. https://doi.org/10.1016/0021-9681(83)90145-5 6885958

[16] Abbott RD , Rodriguez BL, Burchfiel CM, Curb JD. Physical activity in older middle-aged men and reduced risk of stroke: the Honolulu Heart Program. Am J Epidemiol. 1994;139:881–893. https://doi.org/10.1093/oxfordjournals.aje.a117094 8166138

[17] Gordon T , Kagan A, Garcia-Palmieri M, et al Diet and its relation to coronary heart disease and death in three populations. Circulation. 1981;63:500–515. https://doi.org/10.1161/01.cir.63.3.500 7460234

[18] Willcox BJ , Tranah GJ, Chen R, et al The FoxO3 gene and cause-specific mortality. Aging Cell. 2016;15:617–624. https://doi.org/10.1111/acel.12452 27071935 PMC4933667

[19] Willcox BJ , Morris BJ, Tranah GJ, et al Longevity-associated *FOXO3* genotype and its impact on coronary artery disease mortality in Japanese, whites, and blacks: a prospective study of three American populations. J Gerontol A Biol Sci Med Sci. 2017;72:724–728. https://doi.org/10.1093/gerona/glw196 27694344 PMC5964743

[20] Nemtsova V , Vischer AS, Burkard T. Hypertensive heart disease: a narrative review Series—Part 1: pathophysiology and microstructural changes. J Clin Med. 2023;12:2606. https://doi.org/10.3390/jcm12072606 37048689 PMC10094934

[21] Nemtsova V , Burkard T, Vischer AS. Hypertensive heart disease: a narrative review series—Part 2: macrostructural and functional abnormalities. J Clin Med. 2023;12:5723. https://doi.org/10.3390/jcm12175723 37685790 PMC10488346

[22] Donlon TA , Morris BJ, Chen R, et al *FOXO3* longevity interactome on chromosome 6. Aging Cell. 2017;16:1016–1025. https://doi.org/10.1111/acel.12625 28722347 PMC5595686

[23] Zhang W , Zhang S, Yan P, et al A single-cell transcriptomic landscape of primate arterial aging. Nat Commun. 2020;11:2202. https://doi.org/10.1038/s41467-020-15997-0 32371953 PMC7200799

[24] Cao G , Lin M, Gu W, et al The rules and regulatory mechanisms of FOXO3 on inflammation, metabolism, cell death and aging in hosts. Life Sci. 2023;328:121877. https://doi.org/10.1016/j.lfs.2023.121877 37352918

[25] Tothova Z , Kollipara R, Huntly BJ, et al FoxOs are critical mediators of hematopoietic stem cell resistance to physiologic oxidative stress. Cell. 2007;128:325–339. https://doi.org/10.1016/j.cell.2007.01.003 17254970

[26] Tsuchiya K , Westerterp M, Murphy AJ, et al Expanded granulocyte/monocyte compartment in myeloid-specific triple FoxO knockout increases oxidative stress and accelerates atherosclerosis in mice. Circ Res. 2013;112:992–1003. https://doi.org/10.1161/CIRCRESAHA.112.300749 23420833 PMC3736810

[27] Yan P , Li Q, Wang L, et al FOXO3-engineered human ESC-derived vascular cells promote vascular protection and regeneration. Cell Stem Cell. 2019;24:447–461.e8. https://doi.org/10.1016/j.stem.2018.12.002 30661960

[28] Tsuchiya K , Tanaka J, Shuiqing Y, et al FoxOs integrate pleiotropic actions of insulin in vascular endothelium to protect mice from atherosclerosis. Cell Metab. 2012;15:372–381. https://doi.org/10.1016/j.cmet.2012.01.018 22405072 PMC3315846

[29] Tucka J , Yu H, Gray K, et al Akt1 regulates vascular smooth muscle cell apoptosis through FoxO3a and Apaf1 and protects against arterial remodeling and atherosclerosis. Arterioscler Thromb Vasc Biol. 2014;34:2421–2428. https://doi.org/10.1161/ATVBAHA.114.304284 25234814

[30] Yu H , Fellows A, Foote K, et al FOXO3a (Forkhead Transcription Factor O Subfamily Member 3a) Links Vascular Smooth Muscle Cell Apoptosis, Matrix Breakdown, Atherosclerosis, and Vascular Remodeling Through a Novel Pathway Involving MMP13 (Matrix Metalloproteinase 13). Arterioscler Thromb Vasc Biol. 2018;38:555–565. https://doi.org/10.1161/ATVBAHA.117.310502 29326312 PMC5828387

[31] Klinpudtan N , Allsopp RC, Kabayama M, et al The association between longevity-associated *FOXO3* allele and heart disease in septuagenarians and octogenarians: the SONIC study. J Gerontol A Biol Sci Med Sci. 2022;77:1542–1548. https://doi.org/10.1093/gerona/glab204 34254639 PMC9373940

[32] Ahsan T , Urmi NJ, Sajib AA. Heterogeneity in the distribution of 159 drug-response related SNPs in world populations and their genetic relatedness. PLoS One. 2020;15:e0228000. https://doi.org/10.1371/journal.pone.0228000 31971968 PMC6977754

[33] Calissi G , Lam EW, Link W. Therapeutic strategies targeting FOXO transcription factors. Nat Rev Drug Discov. 2021;20:21–38. https://doi.org/10.1038/s41573-020-0088-2 33173189

[34] McIntyre RL , Liu YJ, Hu M, et al Pharmaceutical and nutraceutical activation of FOXO3 for healthy longevity. Ageing Res Rev. 2022;78:101621. https://doi.org/10.1016/j.arr.2022.101621 35421606

